# THE POTENTIAL AND PITFALLS OF TELEHEALTH TO SUPPORT AN AGING POPULATION

**DOI:** 10.1093/geroni/igad104.0474

**Published:** 2023-12-21

**Authors:** Walter Boot

## Abstract

As the population ages, telehealth services are becoming increasingly prevalent and important to help support older adults’ aging in place and manage their health at a distance. However, older adults often face barriers such as technology proficiency, physical and cognitive capacity, and lack of technical support to access and use telehealth. Designers and researchers need to design and evaluate telehealth interventions tailored to older adults’ needs to ensure these technology-based solutions meet their potential. This session will present a variety of technology-based research exploring the development, evaluation, and implementation of telehealth programs and interventions to better support older adults aging in place. L. Moo will present quantitative and qualitative data on challenges veterans experienced with clinical pharmacists’ video visits. L. Adepoju will present factors associated with in-person-only, telemedicine-only, and hybrid healthcare visits among older adults. P. Freddolino will present an evaluation of a telehealth 101 mini-course offered to older adults. F. Abujarad will discuss the development and evaluation of a digital health intervention (VOICES) to increase elder mistreatment identification. Finally, M. Gately will describe a study of person factors and environmental factors on older adults’ capacity to participate in video telehealth sessions. Older adult barriers and areas of improvement in telehealth design will be discussed throughout this session.

